# Interleukin‐4 induces a CD44_high_/CD49b_high_ PC3 subpopulation with tumor‐initiating characteristics

**DOI:** 10.1002/jcb.26607

**Published:** 2018-01-19

**Authors:** Holger H.H. Erb, Fabian Guggenberger, Frédéric R. Santer, Zoran Culig

**Affiliations:** ^1^ Department of Urology and Pediatric Urology University Medical Center Mainz Mainz Germany; ^2^ Division of Experimental Urology, Department of Urology Medical University of Innsbruck Innsbruck Austria; ^3^ Center of Biomolecular and Cellular Engineering International Clinical Research Center, St. Annés University Hospital Brno Brno Czech Republic

**Keywords:** basal cells, cancer stem cells, cytokine, prostate cancer

## Abstract

Pro‐ and anti‐inflammatory cytokines may influence proliferation, migration, invasion, and other cellular events of prostate cancer (PCa) cells. The hyaluronan receptor CD44, which is regulated by Interleukin (IL)‐4, is a prostate basal cell marker. CD44_high_/CD49b_high_ expressing cells have been demonstrated to have tumor‐initiating characteristics. Here, we aimed to analyze the effects of long‐term IL‐4 treatment on CD44/CD49b expression, migration, proliferation, and clonogenic potential of basal‐like PCa cells. To this end PC3 cells were treated over 30 passages with 5 ng/mL IL‐4 (PC3‐IL4) resulting in an increased population of CD44_high_ expressing cells. This was concurrent with a clonal outgrowth of cuboid‐shaped cells, with increased size and light absorbance properties. Flow cytometry revealed that the PC3‐IL4 CD44_high_ expressing subpopulation corresponds to the CD49b_high_ population. Isolation of the PC3‐IL4 CD44_high_/CD49b_high_ subpopulation via fluorescence‐associated cell sorting showed increased migrative, proliferative, and clonogenic potential compared to the CD44_low_/CD49b_low_ subpopulation. In conclusion, IL‐4 increases a PC3 subpopulation with tumor‐initiating characteristics. Thus, IL‐4, similar to other cytokines may be a regulator of tumor‐initiation and hence, may present a suitable therapy target in combination with current treatment options.

## 1 INTRODUCTION

The human prostate gland consists of a bilayer of two main epithelial cell types; basal and luminal cells, as well as a small population of neuroendocrine cells. Prostate adenocarcinoma commonly has a luminal phenotype and has been diagnosed for some time by the absence of basal cells.^1^ However, several studies have demonstrated that a small population of primitive malignant cells with a basal phenotype (characterized by CD44+, AR‐, CD49f+, cytokeratins (CK) 5/14+, and p63+ markers) exist, which have the capacity to recapitulate tumors.^2^, ^3^, ^4^, ^5^

The adhesion molecule CD44 can be spliced into many different isoforms and is involved in multiple cellular signaling functions such as migration, proliferation, differentiation, and survival (reviewed in Ponta et al^6^). Several studies have shown dysregulated CD44 expression in the majority of human cancers, including prostate cancer (PCa). However, the role of the CD44 isoforms in PCa remains under discussion. Iczkowski et al reported an inhibitory effect on invasion, migration, growth counts, and soft agar colony formation by CD44 in vitro (reviewed in Iczkowski et al^7^). The group also reported divergent effects of CD44, depending upon the specific tumor environment. By contrast, Patrawala et al^8^ showed that high CD44 expression was found on tumorigenic and metastatic progenitor prostate cancer cells in vivo.

There is evidence that Interleukin (IL)‐4 regulates CD44 expression in several in vitro and in vivo models through STAT6.^9^, ^10^, ^11^ IL‐4 is a multifunctional cytokine discovered in the mid‐1980s that plays a critical role in the regulation of immune responses.^12^ Several studies have shown that IL‐4 (normally produced by tumor‐infiltrating lymphocytes) is elevated in patients with progressive PCa.^13^, ^14^, ^15^ In vitro studies using PCa cell lines have demonstrated that IL‐4 activates NF‐κB and AR in a ligand‐independent manner.^16^ Treatment of androgen‐sensitive LNCaP cells with IL‐4 increased the expression of the co‐activators CBP/p300 and their histone acetyltransferase activity.^17^, ^18^ Overexpression of IL‐4 has been observed to lead to increased proliferation of LNCaP and 22Rv1 cell lines,^19^ while IL‐4 treatment can also induce the proliferation of the AR‐negative PC3 cells under nutrient‐depletion stress.^20^ In addition, Nappo et al^21^ have shown that IL‐4 increases the clonogenic potential of prostate stem‐like cells by activation of STAT6 signaling.

In this study, we show that IL‐4 treatment resulted in an increased cellular subpopulation demonstrating high CD44 expression in the PC3 basal‐like PCa cell line. These cells showed increased mobility, proliferation, and clonogenic potential compared with cells with a low CD44 expression.

## 2 MATERIALS AND METHODS

### 2.1 Cell lines and culture

PC3 and Du‐145 PCa cells were obtained from the American Type Culture Collection (Rockville, MD), cultured, and authenticated as previously described.^22^ For long‐term treatment cells were passaged once weekly in medium containing FCS and 5 ng/mL IL‐4.

### 2.2 [^3^H] Thymidine incorporation assay and MTT assay

Cells were seeded at a density of 2 × 10^3^ cells/well in triplicates onto 96‐well plates. For [^3^H] Thymidine incorporation 37000 Bq (1 μCi)/well [^3^H] thymidine was added for 24 h prior harvesting. DNA was harvested on 96‐well filter plates (Perkin‐Elmer, Brunn am Gebirge, Austria). Fifty micro liter scintillation fluid was then added, and radioactivity was quantified using a Chameleon 5025 plate reader equipped with a liquid scintillation counter (HVD Life Sciences, Vienna, Austria). For cell viability the MTT kit EZ4U Cell Proliferation Assay (Biomedica, Vienna) was used following the manufacturer's protocol and absorbance was quantified using a Chameleon 5025 plate reader.

### 2.3 Flow cytometry and fluorescence associated cell sorting

For the measurement of the cell surface markers CD24, CD44, and CD49b cultivated cells were harvested with a rubber policeman and washed in PBS. Cells were blocked in FACS buffer containing 6 μg/mL human IgG_1_ (Dinova, Königswinter, Germany) and stained according to the manufacturer's protocol using the antibodies depicted in Supplementary Table S1. Measurement was performed using a BD FACS Calibur flow cytometer (Becton Dickinson). The analysis was performed by Cell Quest software version 4.0.1 (Becton Dickinson). The CD44_low_/CD49b_low_ population (expressing low levels of CD44 and CD49b) and the CD44_high_/CD49b_high_ population (expressing high levels of CD44 and CD49b) were sorted via a FACS Aria cell sorter (Becton Dickinson) using the gating strategy as shown in Supplementary Figure S1.

### 2.4 Cell migration and invasion assays

Cell migration and invasion assays were performed as previously described.^23^

### 2.5 Clonogenic assay

For the clonogenic assay, 100 cells were plated in triplicate onto a 6‐well plate in the presence or absence of 5 ng/mL IL‐4. Colonies were counted after 15 days and recorded if they contained more than 32 cells (equaling to five population doublings).

### 2.6 Microscope imaging

Microscope images were taken using a Zeiss Imager Z2 microscope (Zeiss, Vienna) equipped with a Pixelink PLB622‐CU camera (Canimpex Enterprises Ltd, Halifax, NS, Canada).

### 2.7 Statistical analysis

Prism 7 (GraphPad Software, La Jolla, CA) was used for statistical analyses. Gaussian distribution was determined using Kolmogorov‐Smirnov test. Mann‐Whitney U (non‐Gaussian distribution) or Student's *t*‐test (two‐sided, Gaussian distribution) were used to determine whether two sets of data were significantly different from each other. Data are presented as mean ± s.d. or mean ± s.e.m. unless otherwise specified. Mean ± s.d. was used to describe the distribution of the sample values within an experiment and s.e.m was used to estimate how variable the means were in multiple repeated experiments.^24^ *P* values of ≤0.05 were considered significant. All differences highlighted by asterisks were statistically significant as encoded in figure legends (*P ≤ 0.05; **P ≤ 0.01; ***P ≤ 0.001). All experiments were performed in at least three independent biological replicates.

## 3 RESULTS

### 3.1 Differential effects of long‐term IL‐4 treatment on CD44 expression in PCa cell lines

**Figure 1:**
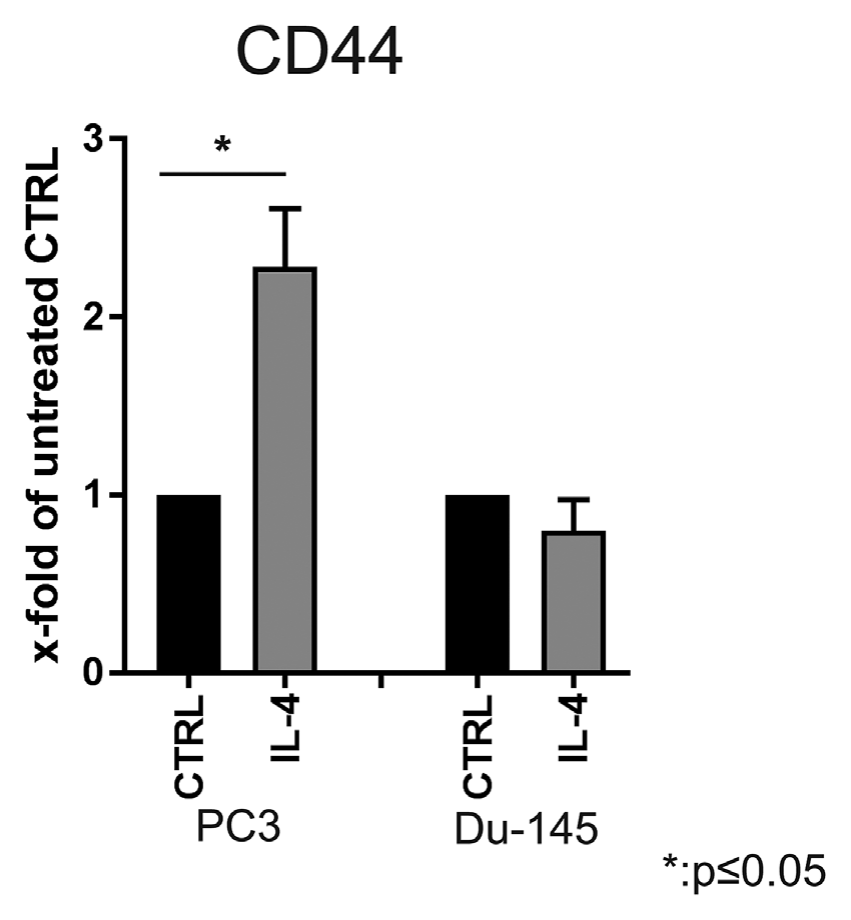
CD44 expression in PC3 and Du‐145 cell lines after long‐term IL‐4 treatment. Flow cytometry analysis of expression of the surface marker CD44. Before staining, PC3 and Du‐145 cells were treated for 20 passages with 5 ng/mL IL‐4 (*n* = 3, *P ≤ 0.05)

It was previously shown that CD44 expression can be regulated by IL‐4/STAT6 signaling.^11^ We therefore measured CD44 expression in the basal‐like prostate cancer cell lines PC3 and Du‐145 using flow cytometry after 20 passages of treatment with 5 ng/mL IL‐4. As shown in Figure 1, CD44 was significantly increased by long‐term IL‐4 treatment in PC3 cells. In contrast, Du‐145 showed no change in CD44 expression. Thus, only PC3 cells were used for the following experiments.

### 3.2 Increase in CD44_high_ expressing cells after long‐term IL‐4 treatment

**Figure 2:**
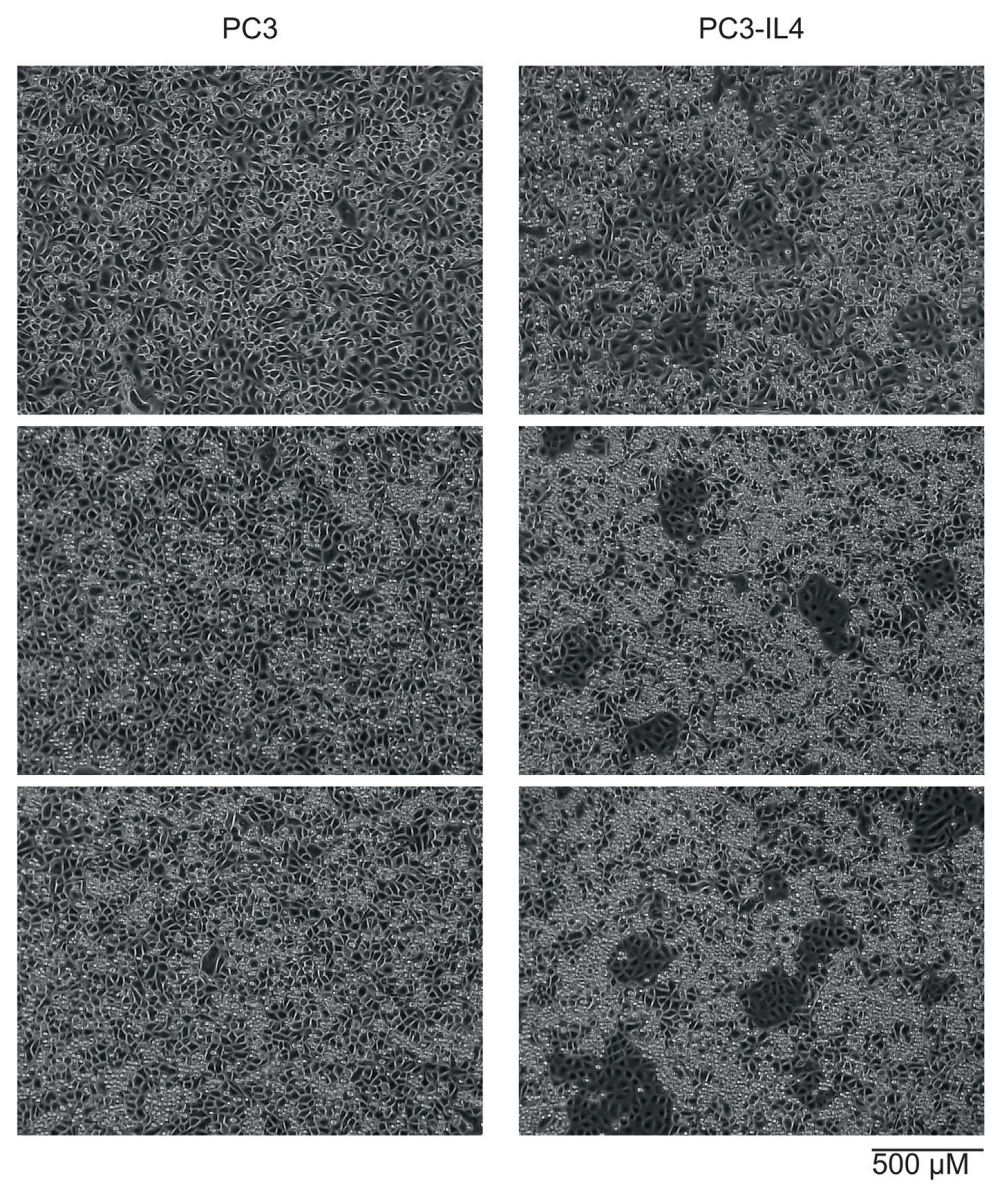
Phase‐contrast analysis of morphological changes in PC3 cells after long‐term IL‐4 treatment. Microscopy images of PC3 and IL‐4 long‐term treated PC3 (PC3‐IL4 cells)

**Figure 3:**
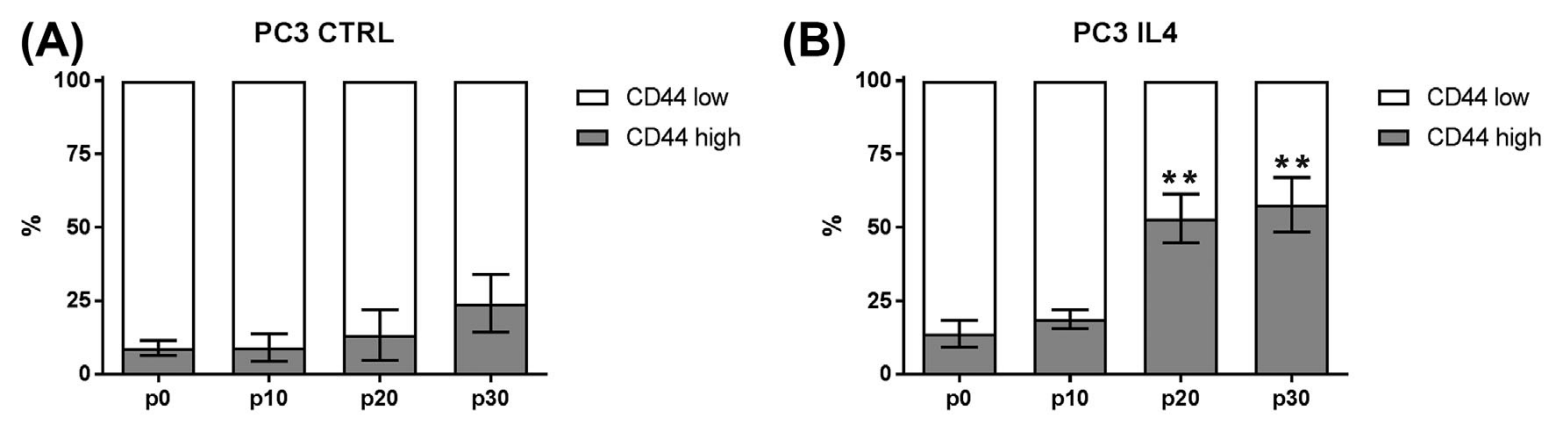
Ratio of CD44_low_ and CD44_high_ subpopulations within the PC3 cell line after long‐term IL‐4 treatment. Ratio of CD44_low_ and CD44_high_ cells in PC3 over 30 passages analyzed by flow cytometry without (A) and with (B) 5 ng/mL IL‐4 treatment (*n* = 3; **P ≤ 0.01)

Morphological changes of PCa cell lines after long‐term cytokine treatment have previously been observed.^25^, ^26^ Microscopic phase‐contrast analysis of the long‐term IL‐4‐treated PC3 (PC3‐IL4) cells revealed clonal outgrowths of cuboid‐shaped cells with increased size and light absorbance properties residing within the morphologically normal PC3 cell population (Figure 2). We consequently tracked the CD44_high_ population during long‐term IL‐4 treatment. The percentage of the CD44_high_ expressing cell subpopulation was significantly increased after 20 passages under IL‐4 treatment (Figure 3B). A similar ratio of CD44_high_ to CD44_low_ expressing cell was observed after 30 passages of IL‐4 treatment. This effect was not seen in untreated PC3 cells (Figure 3A).

### 3.3 Increased CD49b expression in the CD44_high_ subpopulation of PC3 and PC3‐IL4

**Figure 4:**
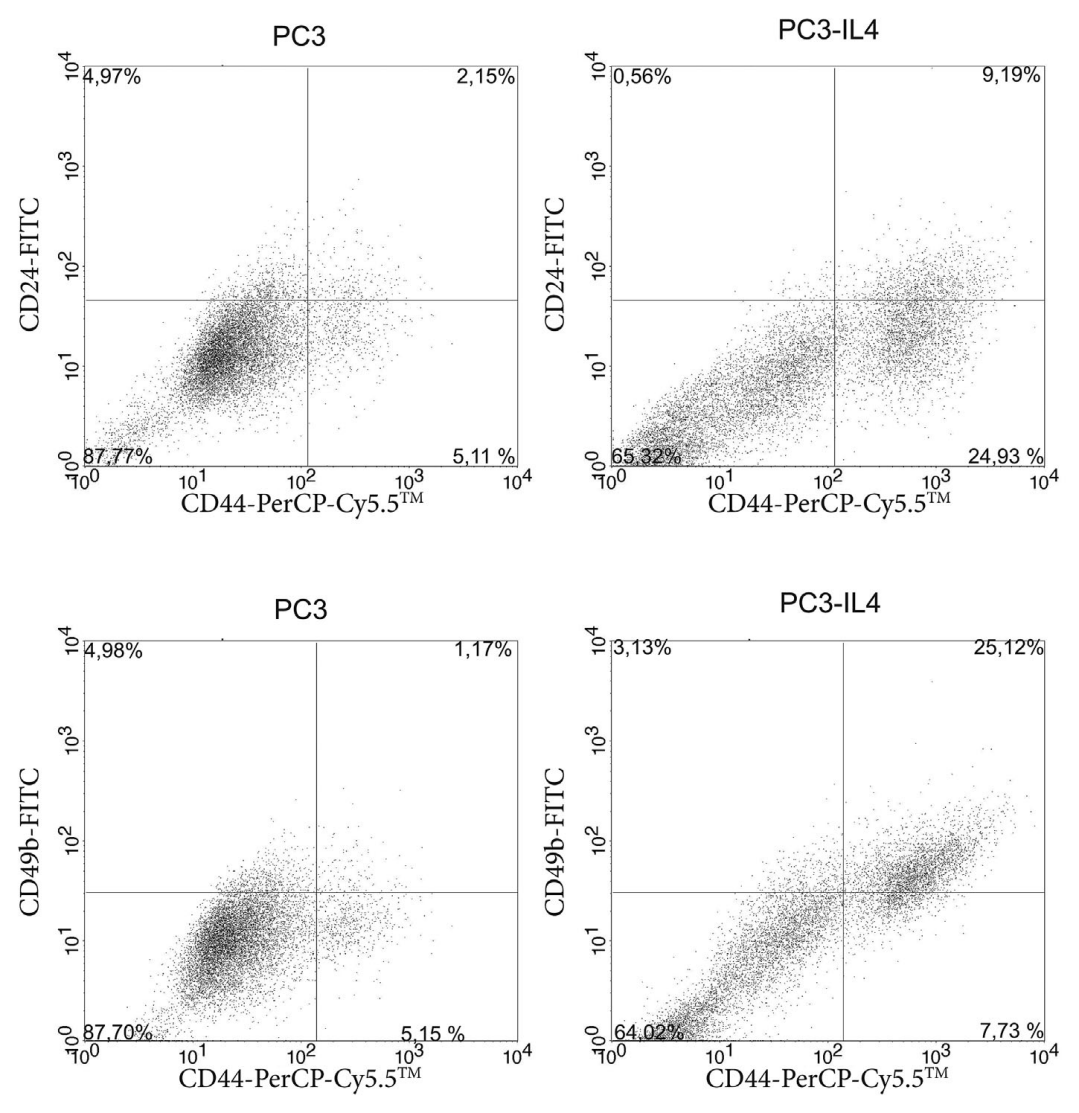
Increased expression of CD49b on CD44_high_ PC3‐IL4 cells. Representative expression analysis of the surface markers CD49b, CD44, and CD24 markers by flow cytometry. (A) CD44 and CD24 staining of PC3 and PC3‐IL4 cells. (B) CD44 and CD49b staining of PC3 and PC3‐IL4 cells

A previously published study from this laboratory showed an increase of a CD44_high_ expressing cell population in docetaxel‐resistant PC3 cells.^27^ This coincided with increased expression of the cancer stem cell (CSC)‐like marker CD49b.^28^ We therefore investigated the expression of CD24 (a marker for low‐differentiated to full‐differentiated, luminal prostate cells^29^, ^30^), CD44, and CD49b after long‐term IL‐4 treatment. Interestingly, PC3 and PC3‐IL4 cells were observed to harbor a population of CD24_low_/CD44_high_/CD49b_high_ cells (Figure 4). Similar to previous results, this population was very small in the parental PC3 cells.^27^ Interestingly, long‐term IL‐4 treatment led to an increase of this population leading to the conclusion that IL‐4 can trigger the growth of a PC3 subpopulation expressing basal‐ and CSC‐like markers. Similar to docetaxel‐resistant PC3 cells,^27^ PC3‐IL4 cells also showed signs of epithelial‐to‐mesenchymal transition with decreased CD324 (E‐Cadherin) and increased CD325 (N‐Cadherin) expression of the PC3‐IL4 CD44_high_ population compared to the CD44_low_ population (Supplementary Figure S2).

### 3.4 Increased migration of the CD24_low_/CD44_high_/CD49b_high_ PC3‐IL4 subpopulation

**Figure 5:**
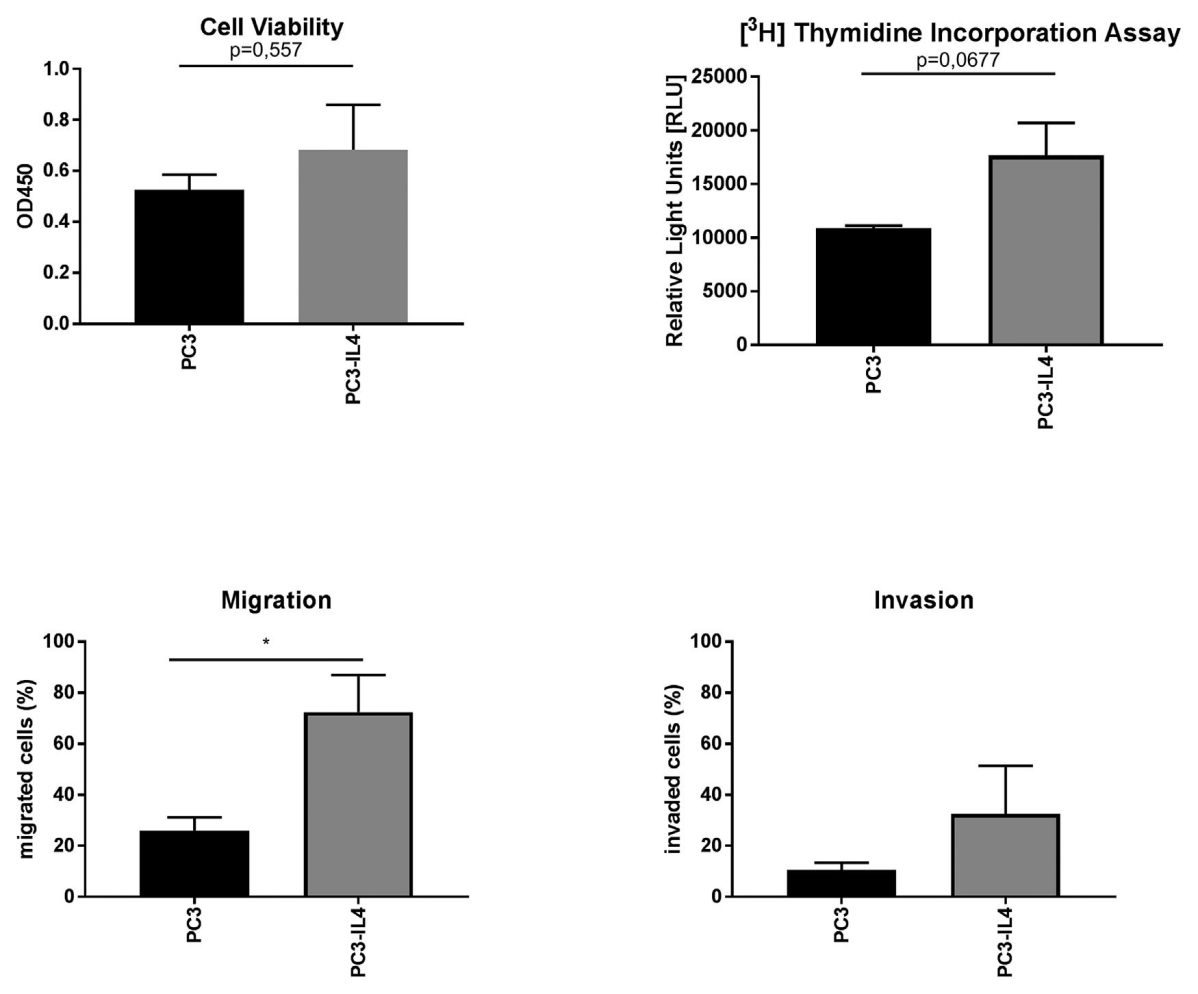
Increased migration of PC3‐IL4 cells. Functional comparison of PC3 and PC3‐IL4 cells. (A) Cell viability was determined by the EZ4U assay. (B) Cellular proliferation was assessed by measurement of [^3^H] Thymidine Incorporation. (C) Migration and (D) invasion through Matrigel were assessed by Boyden Chamber assays (*n* = 3, *P ≤ 0.05)

**Figure 6:**
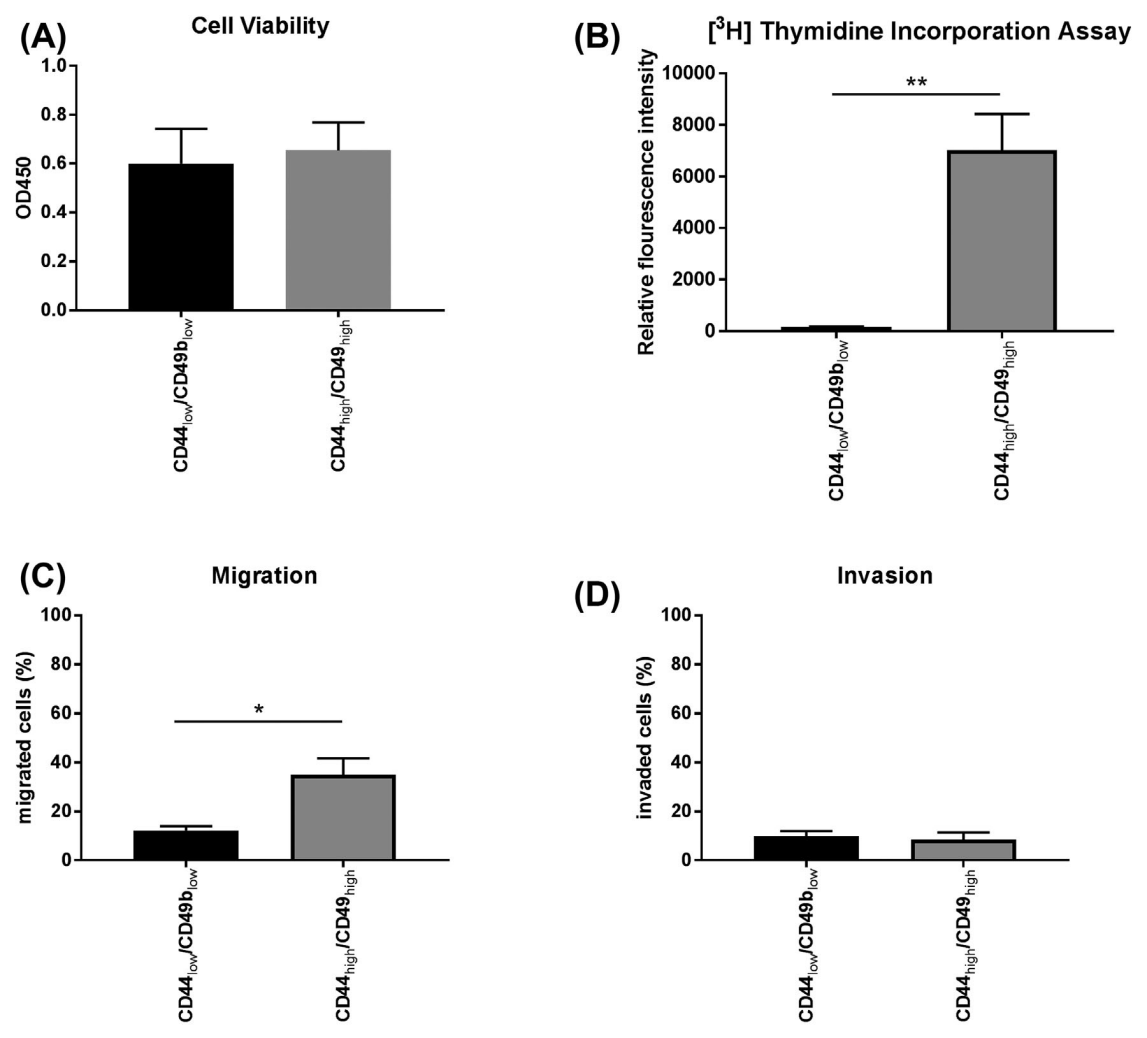
Increased proliferation and migration of the PC3‐IL4 CD44_high_/CD49b_high_ subpopulation. Functional comparison of the PC3‐IL4 CD44_low_/CD49b_low_ and CD44_high_/CD44b_high_ subpopulations. (A) Cell viability was determined by the EZ4U assay. (B) Cellular proliferation was assessed by measurement of [^3^H] Thymidine Incorporation. (C) Migration and (D) invasion through Matrigel were assessed by Boyden Chamber assays (*n* = 3, *P ≤ 0.05, **P ≤ 0.01)

Next, we investigated whether the CD24_low_/CD44_high_/CD49b_high_ population within the PC3‐IL4 cell line has also gained physiological characteristics of malignant basal‐like cells, that is, increased cell mobility and proliferation. Initial results showed that PC3‐IL4 cells have no significant difference in cell viability and proliferation (Figure 5A + B) compared to the untreated PC3. In order to determine whether IL‐4 influenced the chemotaxis of primary PCa cells, migration and invasion assays were performed with Boyden chambers in the presence of 30% FCS as a chemoattractant. PC3‐IL4 showed a significant difference in cell migration (Figure 5C) but no significant change in cell invasion through matrigel (Figure 5D). To investigate if the observed effects were due to the increased CD44/CD49b expression of PC3‐IL4 cells, the CD44_high_/CD49b_high_ subpopulation and the CD44_low_/CD49b_low_ subpopulation were isolated via a FACS Aria Cell Sorter and compared. The cell subpopulations showed no difference in cell viability (Figure 6A). However, there was almost no thymidine incorporation in the CD44_low_/CD49b_low_ population compared to the CD44_high_/CD49_high_ population (Figure 6B). Furthermore, the CD44_high_/CD49b_high_ population showed a significant increase in cell migration (Figure 6C), but again no difference in cell invasion through matrigel could be observed (Figure 6D).

### 3.5 Increased clonogenic potential of the IL‐4 induced CD44_high_/CD49b_high_ population

**Figure 7:**
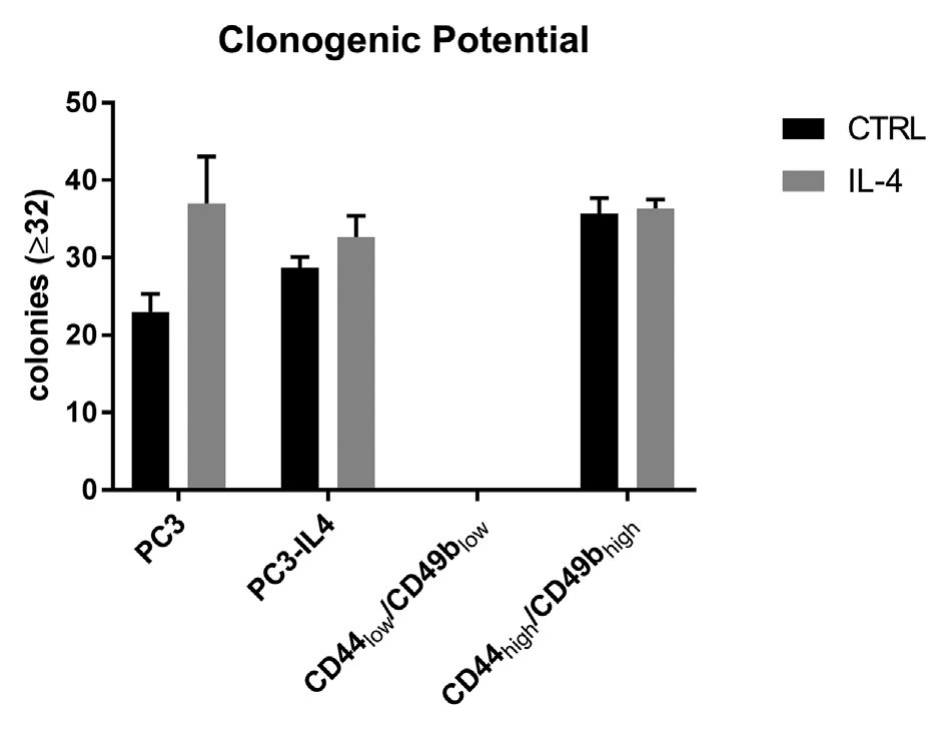
Influence of IL‐4 treatment on the clonogenic potential. Clonogenic assays were performed for PC3, PC3‐IL4, and PC3‐IL4 derived CD44_low_/CD49b_low_ and CD49b_high_/CD44_high_ subpopulations in the presence or absence of 5 ng/mL IL‐4 (*n* = 3)

To further address the physiological properties of the IL‐4 generated CD44_high_/CD49b_high_ population clonogenic assays, as an indicator for tumor‐initiation, were performed. Firstly, clonogenic assays with PC3 and PC3‐IL4 cells were performed in the presence or absence of IL‐4 treatment (Figure 7). Although not significant, PC3 cells showed an increased colony forming efficiency in presence of IL‐4 compared to untreated controls. PC3‐IL4 cells were not affected when IL‐4 was omitted from the medium and showed a slightly higher number of colonies compared to the parental PC3 cells. Next, clonogenic assays were repeated with PC3‐IL4 after sorting based on CD44/CD49b expression. Independent of IL‐4 treatment, the isolated CD44_low_/CD49b_low_ subpopulation was not able to form colonies. However, regardless of IL‐4 treatment the CD44_high_/CD49b_high_ subpopulation demonstrated a similar colony forming efficiency to PC3‐IL4 or parental PC3 cells in presence of IL‐4.

## 4 DISCUSSION

Studies from the last decade have demonstrated the existence of tumor‐initiating cells in PCa.^31^ Increasing data supports the concept of malignancy within the basal layer of the prostate tissue giving rise to luminal adenocarcinoma via aberrant differentiation (reviewed in Strand and Goldstein^32^). Cells within the basal layer are characterized by the absence of AR protein expression and are thus androgen‐independent, yet responsive to androgens to induce differentiation.^33^ The PCa cell lines PC3 and Du‐145 are AR‐negative and may be considered as basal‐like models of PCa.^34^ Although these two cell lines have lost expression of several basal markers, such as p63,^35^ possibly through adaptation to long‐term ex vivo two‐dimensional culture, several characteristics are retained. Indeed, a small subpopulation of basal‐like cells expressing high levels of the basal cell marker CD44 exists within the PC3 cell line. This subpopulation has been demonstrated to be more proliferative, clonogenic, tumorigenic, and metastatic than the subpopulation expressing no/low CD44.^8^ Furthermore, the CD44_high_ expressing subpopulation was increased in docetaxel‐resistant PC3 showing an involvement in therapy resistance.^27^ Here, we demonstrate that long‐term treatment with the cytokine IL‐4 is also able to increase the number of the CD44_high_ subpopulation within the PC3 cell line (Figure 3). In addition, this subpopulation shows characteristics of CSC‐like cells such as increased proliferation, migration, and clonogenic potential compared to the CD44_low_/CD49b_low_ subpopulation, as well as markers of epithelial‐to‐mesenchymal transition (Figures 6B + C, 7, and Supplementary S2). IL‐4 is a direct regulator of CD44 via its downstream transcription factor STAT6.^11^ While we found that IL‐4 long‐term treatment was indeed sufficient to increase CD44 by 2.5‐fold in PC3 cells (Figure 1), this could not be observed in Du‐145. An explanation for this could be the fact that Du‐145 contain a heterozygous frameshift in the STAT6 gene (p.Q281fs*92) as revealed by analysis of the COSMIC database.^36^ This frameshift precludes expression of the JAK phosphorylation residue as well as protein interaction domains such as SH2 and the LXXLL motif. This is also in line with Du‐145 lacking the ability to bind the extracellular matrix glycosaminoglycan hyaluronan, the ligand for CD44.^37^ However, a thorough analysis of STAT6 signaling in Du‐145 is warranted in order to draw conclusions. Altogether, we conclude that IL‐4 is a regulator of basal‐like cells showing characteristics of tumor‐initiating cells by inducing expression of CD44.

Similar to normal stem cells regulated by their niche, tumor‐initiating cells are regulated by the tumor microenvironment by a complex network of cytokines and growth factors (for review Korkaya et al^38^). Stem cell regulatory pathways that are frequently dysregulated in cancer include Notch, Hedgehog, Wnt, PI3K, and Jak/STAT. Previously, the importance of IL‐4 as a regulator of tumor‐initiating/cancer stem cell (CSC)‐like cells has been documented in several cancer types. In colon cancer, CD133‐positive tumor‐initiating cells are autocrine for IL‐4^39^ and upregulate the anti‐apoptotic and STAT‐6 target Survivin.^40^ In breast cancer cells an antagonist of the IL‐4 receptor IL4Rα was able to reduce the number of CD44^+^/CD24^−^ CSC‐like cells.^41^ Similarly, knockdown of IL4Rα in the pancreatic cancer cell line Capan‐1 resulted in reduced cell growth, anchorage‐independent colony size and inhibition of migration.^42^ Previously, we were able to show an involvement of IL‐4 in the clonogenic potential of prostate stem‐like cells isolated from PCa tissues.^21^ Thus, similar to other cytokines and chemokines, such as IL‐6 and ‐8, IL‐4 is a factor with potential to regulate tumor‐initiating/CSC‐like cells and thereby promote tumor progression and possibly metastases formation.

IL‐4 plasma concentrations of castration‐resistant PCa patients were found elevated compared to treatment‐naïve patients.^43^ Moreover, cytokine profiling revealed that IL‐4, among other cytokines, was increased and associated with progressive disease after the first treatment cycle with docetaxel.^44^ In contrast, administration of IL‐4 to patients with chronic lymphocytic leukemia provoked progressive disease during treatment attributable to an increase in lymphocyte blood count.^45^ It can therefore be speculated that IL‐4 or its downstream signaling cascade, in particular STAT6, could be a therapeutic target. This and previously mentioned studies predict highest effects of an IL‐4 signaling blockade on tumor‐initiating/CSC‐like cells leading to the conclusion that an anti‐IL‐4/STAT6 therapy may have considerable potential in combination with a therapy that is directed against the bulk of tumor cells. However, inhibitors of the IL‐4 cascade such as, for example, the STAT6 inhibitor AS1517499 are in an early pre‐clinical development for immunological disorders.^46^ Furthermore, more work is necessary to fully understand the role of IL‐4/STAT6 in regulation of CSC‐like cells and confirm this signaling axis as a target for cancer therapy.

## 5 CONCLUSIONS

In summary, we could show that IL‐4 is a potent inducer of the CD44_high_/Cd49b_high_ subpopulation residing within the PC3 cell line. This subpopulation shows characteristics of tumor‐initiating cells such as increased migrative, proliferative, and clonogenic potential.

## DISCLOSURE

The authors declare no conflict of interest.

## Supporting information

Additional Supporting Information may be found online in the supporting information tab for this article.

**Figure S1**. Gating Strategy for sorting CD44_low_/CD49b_low_ and CD44_high_/CD49_high_ cells.
**Figure S2**. Expression of E‐cadherin (CD324) and N‐Cadherin (CD325) of CD44_low_ and CD44_high_ PC3‐IL4 cells.
