# Supplementary material for: Interleukin‐4 induces a CD44high/CD49bhigh PC3 subpopulation with tumor‐initiating characteristics
Source: J Cell Biochem. 2018 Jan 19;119(5):4103–12. doi: 10.1002/jcb.26607 (PMC5900863; doi:10.1002/jcb.26607)

| **Name** | **Company** |
| --- | --- |
| PerCP-Cy™5.5 Mouse Anti-Human CD44 | Becton Dickinson, Heidelberg, Germany |
| PerCP-Cy™5.5 Mouse IgG2b, κ Isotype Control | Becton Dickinson, Heidelberg, Germany |
| FITC Mouse Anti-Human CD49b | Becton Dickinson, Heidelberg, Germany |
| FITC Mouse IgG1, κ Isotype Control | Becton Dickinson, Heidelberg, Germany |
| FITC Mouse Anti-Human CD24 | Becton Dickinson, Heidelberg, Germany |
| Alexa Fluor 488 Anti-Human CD324 | Becton Dickinson, Heidelberg, Germany |
| Alexa Fluor 488 Anti-Human CD325 | Becton Dickinson, Heidelberg, Germany |


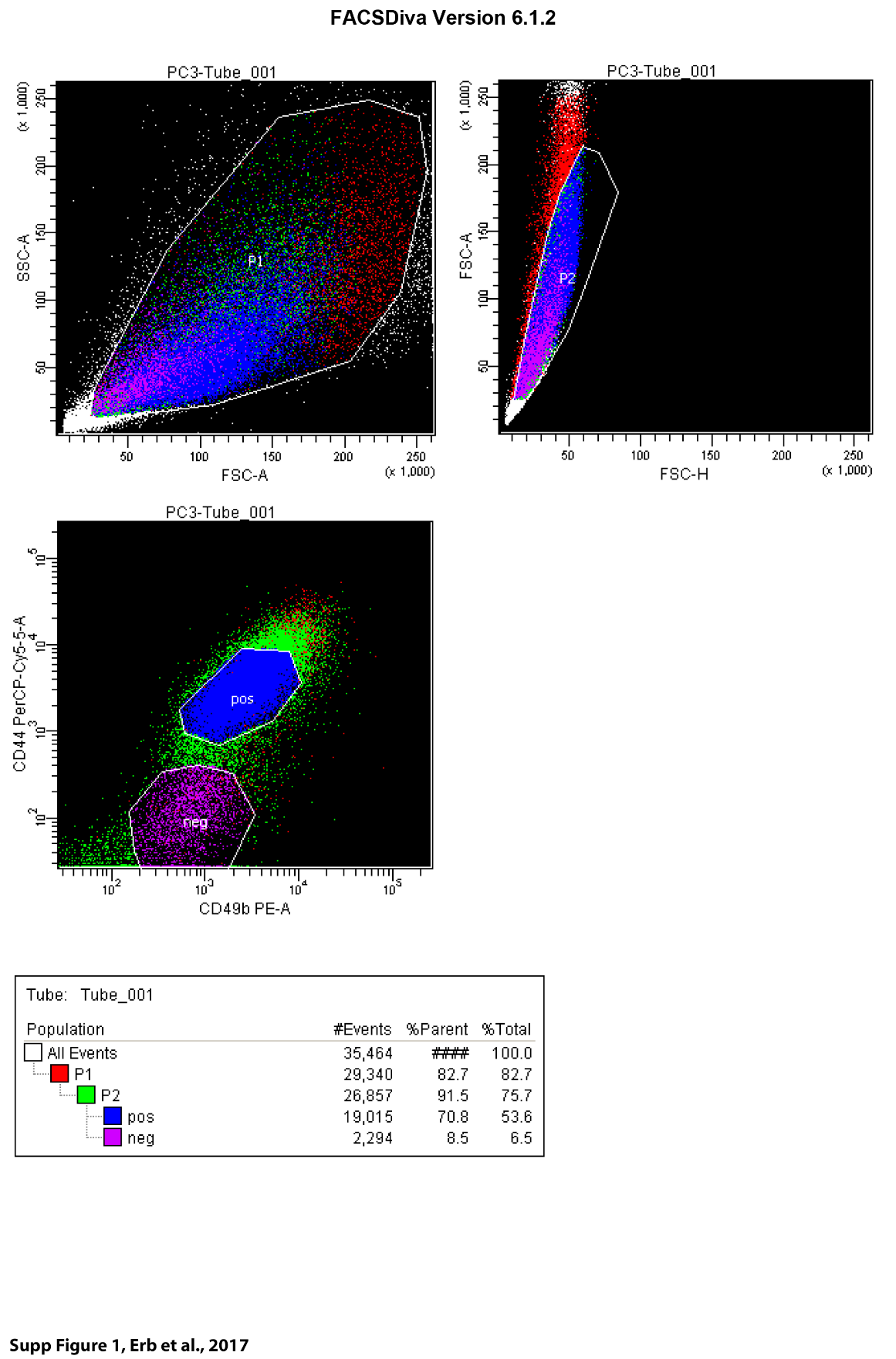


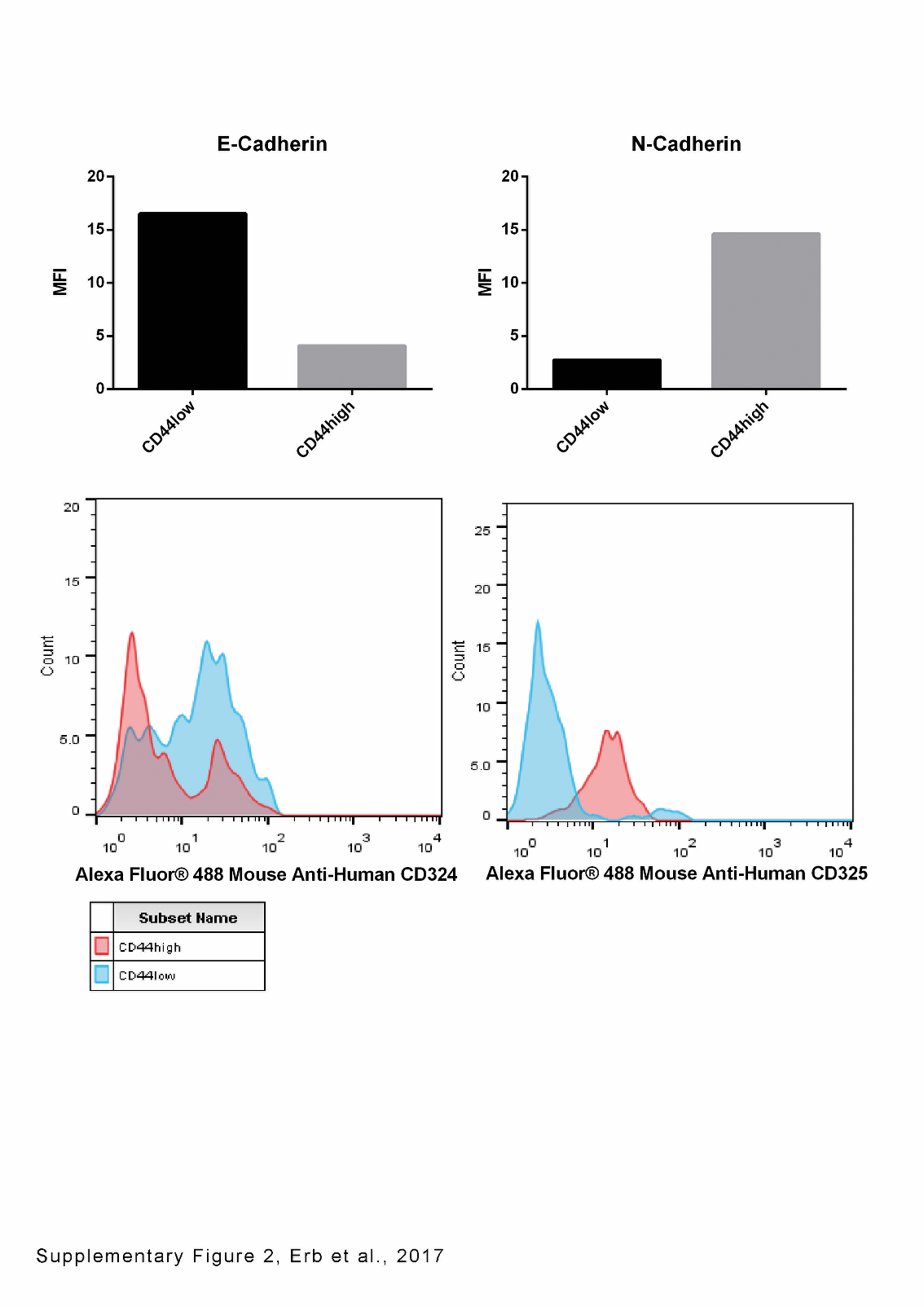

Supplement: Supplementary file 1 — Figure S1. Gating Strategy for sorting CD44low/CD49blow and CD44high/CD49high cells. Figure S2. Expression of E‐cadherin (CD324) and N‐Cadherin (CD325) of CD44low and CD44high PC3‐IL4 cells. [file JCB-119-4103-s001.docx]
